# Musculoskeletal pain and associated factors among Ethiopian elementary school children

**DOI:** 10.1186/s12891-018-2192-6

**Published:** 2018-07-31

**Authors:** Manayesh Delele, Balamurugan Janakiraman, Abey Bekele Abebe, Ararso Tafese, Alexander T. M. van de Water

**Affiliations:** 1 Department of Physiotherapy, School of Medicine and Health Sciences, University of Gondar and Gondar University specialized comprehensive hospital, Gondar, Ethiopia; 2 Department of Public Health, School of Medicine and Health Sciences, University of Gondar, Gondar, Ethiopia; 3 School of Physiotherapy, Academy of Health, Saxion University of Applied Sciences, Enschede, The Netherlands

**Keywords:** Musculoskeletal pain, School children, School bag, Walking distance, Ethiopia

## Abstract

**Background:**

Ethiopian school children often carry school supplies in heavy school bags and encounter limited school facilities. This stresses their vulnerable musculoskeletal system and may result in experiencing musculoskeletal pain. High prevalence of musculoskeletal pain has been documented, but data on musculoskeletal pain among elementary school children in Ethiopia is lacking. To determine the prevalence of musculoskeletal pain and associated factors among elementary school children in Gondar, Ethiopia.

**Methods:**

Cross-sectional study was conducted among children from six randomly selected elementary schools. Sample size was determined proportionally across school grades and governmental and private schools to ensure variety within the sample. Data collection consisted of physical measurements including height, weight and schoolbag weight, and a structured questionnaire on musculoskeletal pain, mode of transport, walking time and school facilities. Data were analysed descriptively and through uni- and multivariate logistic regression model.

**Results:**

In total 723 children participated. The overall prevalence of self-reported musculoskeletal pain was 62%, with a significant difference between school types (governmental 68% versus private 51%). Shoulder, neck and lower leg/knee were most commonly reported. Walking to and from school for ≥20 min (OR = 2.94, 95% CI 2.05 to 4.21) and relative school bag weight (OR = 2.57, 95% CI 1.48 to 4.47) were found significantly associated with self-report musculoskeletal pain. Children with carrying heavy school supplies and also walking long duration have a 3.5 (95% CI = 1.80–6.95) times greater chance of reporting pain as compared to those who carry lesser weighed bags and reported shorter walking duration at the same time.

**Conclusions:**

Prevalence of self-reported musculoskeletal pain was high among children attending public schools and also those who walked a long way to and from school. Long walking duration and relative school bag weight were significantly associated with musculoskeletal pain. These findings can inform policymakers to provide transportation services and other facilities at elementary schools. The findings of this study should be interpreted with caution due to possible social desirability bias with higher prevalence of self-reported pain and more so in children population.

## Background

Musculoskeletal pain among school-aged children is a well-known concern as acknowledged by WHO interdisciplinary experts studying school environments [1]. The overall lifetime prevalence of musculoskeletal symptoms among school children in developed countries ranges from 16 to 86% [2–4] and in developing countries these figures are higher, ranging from 46.3 to 88.8% [5–9].

Ethiopia is one of the fastest developing countries in Africa. There is a sustained increase in the number of both public and private schools and the educational system is evolving. The expanding school syllabus has resulted in children having to carry more school supplies, while many Ethiopian schools still have low resource facilities lacking to offer student lockers in schools and transport coverage to and from schools [10, 11]. Although some researchers debate that there is still lack of evidence on the short and long-term effects of determinants of musculoskeletal pain among school children [12], others report that children’s developing musculoskeletal system is negatively influenced by factors such as heavy school bags, lack of locker facilities, walking to and from school, sitting postures, method of carrying school supplies, body mass index and furniture facilities [13–15]. For example, excessive school bag weight could in the long term result in deteriorating biomechanical effects on the rapidly growing musculoskeletal system of young aged children. These findings formed the basis for recommended school bag weight limits. Professional associations advise that school children should carry no more than 15 to 20% of their bodyweight [16–18].

Although implied, literature searching revealed that the burden of musculoskeletal pain among school children and its associations have not been established or explored in Ethiopia. Considering the additional burden of child health issues and potential long-term effects of musculoskeletal pain in children on communities and society in a developing country like Ethiopia, it is important to gain an understanding of the extent of this problem. Therefore, this study aimed to investigate the prevalence and to determine the associated factors of musculoskeletal pain among private and public elementary school children in Gondar city, Ethiopia.

## Methods

### Study setting

An institution-based cross-sectional descriptive study was carried out from February 2016 to June 2016. The study was done at 43 public and 21 private elementary schools in Gondar city (300,000 inhabitants, estimated), which is located 741 km North West of Addis Ababa, Ethiopia. Informed consent was obtained from parents/caregivers and school teachers, and assent was obtained from participating children. Ethical approval was obtained from the Gondar University School of Medicine research and ethical review committee (SOM/047/7/08).

### Study participants

The population of interest comprised of all elementary school children’s of both sexes, aged 18 or less and living in Gondar city. Inclusion criteria were parental consent, children assent, ability to ambulate independently and ability to wear school bag while standing. Children with known congenital or structural deformities or recent surgery (within 3 months) were excluded.

### Sample size determination

In total 47,286 elementary school children were registered as student in private and government schools in Gondar city by the local government school authority bureau. The required sample size was calculated using Epi Info software version 7.0 (Centres for Disease Control and prevention, USA) and was based on this registered population. The following assumptions were used to determine the sample size based on single population proportion: prevalence of 50% since no past regional data exist, confidence level of 95%, design effect of 2. The derived sample size was *n* = 768. Accounting for an estimated non-response or refusal rate of 10%, the required sample size was *n* = 845.

### Sampling procedure

A multistage sampling was implemented. The schools were stratified into governmental and private elementary schools. In each strata, schools were proportionally selected based on random selection. Within the selected school, the samples were proportionally allocated based on the total number of children in that school and from each grade, randomly from grade 1 to 8 using their alphabetically ordered list. Children were sampled based on the proportion of gender in the selected grade of the randomly selected six schools.

### Data collection procedure and materials

Prior to data collection, a one-day intensive training was given to data collectors (community-based rehabilitation workers). A pre-test of data collection was carried out with 42 elementary school children from one Gondar city school, prior to actual data collection. Those children were excluded from participation in the main study. Modifications and corrections of the measurement procedures were made based on analyses of pre-test data.

The data collection process was supervised by the principal investigator (MD) on a daily basis to ensure accuracy, completeness and consistency. Consequently, amendments and corrections were made before the start of the next working day.

Elementary school children, who had informed consent from their teacher and a parent/caregiver and assented, were enrolled in the study. First, height of the children was measured to the nearest 1 cm using a stadiometer, and weight was measured using a digital weighing scale (Electrolux, Korea) to the nearest 1 kg. The children, dressed in school uniforms, were instructed to remove their shoes before measuring weight. Weight was initially measured with children standing on the weighing scale with their school bag and then without their school bag. The difference between the two recorded weights was recorded as weight of the school bag. Recalibration of the weighing scale was done after each measurement. Since children carry a lunch box and water bottle, the data were collected only in morning sessions. Body Mass Index (BMI) was calculated, adjusted for children and categorised (https://www.cdc.gov/healthyweight/assessing/bmi/childrens_bmi/about_childrens_bmi.html, [19]).

We used a structured questionnaire to record demographic data such as age, gender and grade level, and associated factors of musculoskeletal pain such as type of school bag, mode of transport, walking time to school, locker facilities, type of furniture at school, time spent sitting, time of physical education. Musculoskeletal symptoms in different body regions were assessed using an Amharic translation of a modified version of the Standardised Nordic questionnaires for musculoskeletal symptoms [20]. The questionnaire includes a body map to allow children to report musculoskeletal pain by labelling the body location and a happy-face sad-face visual pain scale for pain intensity was used. Four rating categories (never, occasionally, frequently and every day) were used to record presence of musculoskeletal pain/symptoms. Care was taken by the data collectors to simplify the questions as much as possible, accompany parents or class teacher during pain reporting and explanations were given whenever questions arose.

### Data analysis

Data were coded and entered into Epi Info software version 7.0 and IBM Statistical Package for Social Sciences (SPSS) version 24 for Windows for statistical analyses. Data entry with the original data was done by the data collector and the main investigator (MD) supervising each other to enhance correctness. In addition, the data was checked by two other researchers (AB and AvdW) for completeness, accuracy and clarity. Descriptive statistics (frequencies, percentages, means and standard deviations (SD)) were used for all participant characteristics and associated factors of musculoskeletal pain.

With musculoskeletal pain (categories: none versus present) as dependent variable, bivariate and multivariate binary logistic regression analyses were executed to examine the association with different independent variables. Independent variables included in the regression models were, age (categorised 5–10, 11–15 and > 15), BMI (categorised underweight, normal weight, overweight and obese), type of school (governmental and private), mode of transport (walking and motorised transport), walking duration (categorised no walking, < 20 min and ≥ 20 min), way of carrying school supplies, percentage of school bag’s weight of body weight (categorised 0–10%, 10–20, > 20%). Multiple regression and interaction terms were used to examine the potential association between school bag weight and musculoskeletal pain differed by hypothesized variables, including gender, type of school, and walking duration. Variables were inputted into the model using forced entry and categories were used as covariates for detailed analyses. Results were considered statistically significant when 95% confidence intervals not containing unity (equal to *p-*value < 0.05) for both main effects and interaction terms. Initially, bivariate analyses were conducted and independent variables that were found statistically significant were included in multivariate analysis. When clear subgroups seemed present in the data, significance testing (Pearson χ^2^) and, if appropriately sized subgroups per category remained, logistic regression were performed.

## Results

### Sample characteristics

Out of 845 consent forms dispatched to six elementary schools in Gondar city, 723 parents (85.6%) consented for their children to participate. This is 94.1% of the power calculated sample size (*n* = 768). The 723 elementary school children were from four governmental schools (*n* = 497; 68.9%) and two private schools (*n* = 226; 31.1%). The mean age of the participants was 11.5 years (SD 2.7 years) and 58.5% (*n* = 423) was female. A normal weight children adjusted BMI was recorded in 524 (72.5%) children, whereas 148 (20.5%) were found being underweight. More sample characteristics are presented in Table 1.

**Table 1:** Sample characteristics and distribution of musculoskeletal pain among school children, Ethiopia

Variables	Sample totals		Musculoskeletal pain			
			Yes		No	
	n	(%)	n	(%)	n	(%)
All participants	723	(100%)	451	(62.4%)	272	(37.6)
Age (in years)						
5–10	272	(37.6%)	173	(23.9%)	99	(13.7%)
11–15	397	(54.9%)	238	(32.9%)	159	(22.0%)
> 15	54	(7.5%)	40	(5.5%)	14	(1.9%)
Sex						
Male	300	(41.5%)	182	(25.2%)	118	(16.3%)
Female	423	(58.5%)	269	(37.2%)	154	(21.3%)
Height (in cm) as mean (SD)	133.1	(13.7)	131.9	(13.2)	135.1	(14.4)
Weight (in kg) as mean (SD)	30.4	(8.7)	29.7	(8.5)	31.5	(8.9)
BMI children adjusted percentiles						
Underweight < 5%	148	(20.5%)	98	(13.6%)	50	(6.9%)
Healthy weight 5–85%	524	(72.5%)	324	(44.8%)	200	(27.7%)
Overweight 85–95%	41	(5.7%)	24	(3.3%)	17	(2.4%)
Obese 95%	10	(1.4%)	5	(0.7%)	5	(0.7%)
Type of school						
Governmental	497	(68.9%)	336	(46.5%)	161	(22.3%)
Private	226	(31.3%)	115	(15.9%)	111	(15.4%)
School grade						
Grade 1–4	380	(52.6%)	240	(33.2%)	140	(19.4%)
Grade 5–8	343	(47.4%)	211	(29.2%)	132	(18.3%)
Mode of Transport						
Walking	546	(75.5%)	368	(50.9%)	178	(24.6%)
School bus	115	(15.9%)	49	(6.8%)	66	(9.1%)
Public transport	14	(1.9%)	6	(0.8%)	8	(1.1%)
Private transport	48	(6.6%)	28	(3.8%)	20	(2.8%)
Method of carrying school supplies						
Backpack	587	(81.2%)	363	(50.2%)	224	(31.0%)
Single strap	93	(12.9%)	58	(8.0%)	35	(4.8%)
Handbag	6	(0.8%)	2	(0.3%)	4	(0.6%)
Without bag in hand	37	(5.1%)	28	(3.9%)	9	(1.2%)
Preference of backpack carrying	(*n* = 587)					
Right side	10	(1.7%)	9	(1.5%)	1	(0.2%)
Left side	2	(0.3%)	2	(0.3%)	0	(0%)
Both shoulder together	575	(98.0%)	352	(48.7%)	223	(30.8%)
Preference of single strap carrying	(*n* = 93)					
Right shoulder	64	(68.8%)	41	(44.1%)	23	(24.7%)
Left shoulder	18	(19.4%)	10	(10.8%)	8	(8.6%)
Alternatively(Left/Right)	11	(11.8%)	7	(7.5%)	4	(4.3%)
Walking duration						
No walking	177	(24.5%)	83	(11.5%)	94	(13.0%)
< 20 min	50	(6.9%)	16	(2.2%)	34	(4.7%)
≥ 20 min	496	(68.6%)	352	(48.7%)	144	(19.9%)
Bag weight in % of bodyweight						
0–10%	250	(34.6%)	142	(19.6%)	108	(14.9%)
11–20%	380	(52.6%)	240	(33.2%)	140	(19.4%)
> 20%	93	(12.9%)	69	(9.5%)	24	(3.3%)

### Musculoskeletal pain

**Fig. 1:**
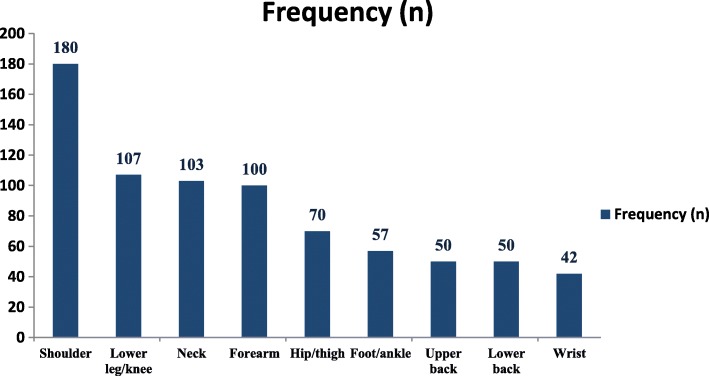
Prevalence of self-reported musculoskeletal pain per body segments

Four hundred and fifty one (*n* = 451, 62.4%) students reported to have experienced musculoskeletal pain in the previous 12 months. Reported prevalence of musculoskeletal pain was nearly equal for female (63.6%, *n* = 269) as for male (60.7%, *n* = 182) participants. A significant difference was observed in musculoskeletal pain prevalence between type of schools (private 50.9% versus governmental 67.6%; χ^2^ (1, *n* = 723) =17.8, *p* < 0.001, *phi* = − 0.16). Most participating children who reported musculoskeletal pain felt this in one (66.2%, *n* = 294) or two body regions (25.7%, *n* = 114). The most frequently recorded regions of experienced musculoskeletal pain was the shoulder (24.9%, *n* = 180) and the least reported musculoskeletal pain region was wrist (5.8%, *n* = 42). An overview of the prevalence of all regions is presented in Fig. 1.

The pain intensity of the majority of participating children who reported musculoskeletal pain was moderate (*n* = 352, 78.1% of 451). Forty-one participants (9.1%) reported severe intensity pain and 58 (12.9%) had mild intensity pain.

### Associated factors of musculoskeletal pain

Walking was the main mode of transport to and from school (75.5%, *n* = 546) followed by a school bus (15.9%, *n* = 115). Mode of transport between type of schools was vastly different (χ^2^ (3, *n* = 723) = 490.4, *p* < 0.001, *phi* = 0.82), with most children from private schools going by school bus (*n* = 113, 50%) or other motorised transport (total *n* = 172, 77.0% versus 23.0%) and nearly all children from governmental schools going by foot (*n* = 494, 99.4% versus 0.6%).

Of those who walked, 68.6% (*n* = 496) walked to school for 20 min or more, which constitutes a subgroup in which a greater prevalence of musculoskeletal pain was found (48.7%, *n* = 352). The school supplies carried by children in both governmental and private schools weighted a mean of 3.84 kg (SD 1.57, range 0-14 kg) and were most commonly carried in a backpack was (81.5%, *n* = 589). Considerable weight (11–20%) was carried by 380 children (52.6%) and 93 children (12.9%) carried supplies weighing > 20% of body weight.

Other relevant factors potentially related to musculoskeletal pain showed no variance between grades or schools. All participants sat more than 330 min per day at school and had less than 100 min of physical education per week. Schools provided no locker facilities for their students’ school supplies.

#### Regression analysis

Prior to analysis, eleven variables potentially related to musculoskeletal pain were identified for regression analysis: age, BMI, type of school, type of school by school bag weight, school bag weight by gender, type of school by duration of walking, mode of transport, duration of walking, duration of walking by school bag weight, method of carrying of school supplies, and relative school bag weight. Of those, age, BMI method of carrying school supplies and type of school by school bag weight in % of body weight were found not significantly associated in univariate analyses (Table 2). The remaining six variables were significant (Table 2) and were evaluated prior to multivariate analysis. Type of school (private or governmental) was significantly related to mode of transport (motorised or walking) and, therefore, both related to walking duration (no walking, < 20 min, ≥ 20 min) violating the assumption of collinearity. Also, multivariate testing revealed that type of school and mode of transport were not significant when adjusting for other included variables and were subsequently removed from the analysis.

**Table 2:** Results of univariate logistic regression of factors associated with musculoskeletal pain among school children, Ethiopia

Variable	Crude Odds ratio	95% CI interval		P
		Lower	Upper	
Age (5–10 years)				
10–15 years	0.86	0.62	1.18	0.34
> 15 years	1.64	0.85	3.15	0.14
Bag/body weight (0–10%)				
10–20%	1.30	0.94	1.81	0.11
> 20%	2.19	1.29	3.71	0.00
BMI (underweight)				
Normal	0.83	0.56	1.21	0.33
Overweight	0.72	0.36	1.46	0.36
Obese	0.51	0.14	1.85	0.31
Carrying supplies (back pack)				
Single strap	1.02	0.65	1.61	0.92
Hand bag	0.31	0.06	1.70	0.18
In hand (no bag)	1.92	0.89	4.14	0.10
Mode of transport (walking)				
Motorized	0.43	0.30	0.60	0.00
Type of school (private)				
Governmental	2.01	1.46	2.78	0.00
Walking duration (no walking)				
< 20 min	0.53	0.27	1.04	0.06
≥ 20 min	2.77	1.95	3.94	0.00
Bag/body weight X Gender	1.138	0.83	1.56	0.42
Bag/body weight X Type of school	1.061	0.71	2.45	0.89
Bag/body weight X Walking duration	5.761	2.67	9.41	0.00
Type of school X walking duration	4.27	1.93	9.44	0.00

The final multivariate regression model (Table 3) included the categorised variables walking duration and relative school bag weight for main effects, type of school by walking duration and school bag weight by walking duration for interaction effects. It was statistically significant (Model χ^2^ (4, *n* = 723) = 65.21, *p* < 0.001), explained 9.1 to 12.5% of variance and classified 66.8% of cases correctly. A non-significant finding was that walking for < 20 min may be protective of musculoskeletal pain as explained by the coefficient below 1 in the logistic regression model, but that walking for ≥20 min significantly increases the odds of musculoskeletal pain by almost 3 (OR = 2.94, 95%CI 2.05 to 4.21). The model also showed that the heavier the schoolbag the more likely it is to have musculoskeletal pain. Compared to 0–10%, the odds increase by 1.5 if the bag weights 10–20% of body weight, and 2.6 if the bag weights > 20% of body weight (Table 3). The interaction effect between heavier school bag weight expressed in percentage of body weight and longer walking duration was also significant (AOR = 3.53, 95% CI: 1.80–6.95, *p* < 0.001).

**Table 3:** Multivariate logistic regression predicting likelihood of musculoskeletal pain among school children, Ethiopia

Variable	Adjusted Odds ratio	95% CI interval		P
		Lower	Upper	
Walking duration < 20 min	0.53	0.27	1.04	0.07
Walking duration ≥20 min	2.94	2.05	4.21	0.00
Bag/body weight 10–20%	1.46	1.04	2.06	0.03
Bag/body weight > 20%	2.57	1.48	4.47	0.00
Bag/body weight X Walking duration	3.534	1.80	6.95	0.00
Type of school X walking duration	0.64	0.29	1.37	0.24
Constant	0.62			0.17

Note: R^2^ = 0.091 (Cox & Snell), 0.125 (Nagelkerke). Model χ^2^ (4, n = 723) =65.21, *p* < 0.001. Correctly predicted 66.8%

Given the significant difference between school types on transportation, subgroup regression analysis (splitting data on private and government schools) was considered. Although, the same variables (walking ≥20 min, relative school bag weight) remained significant in both subgroup models, group sizes in certain categories were too small for adequate analysis.

## Discussion

This is the first study that investigated prevalence of musculoskeletal pain and associated factors among elementary school children in Ethiopia. The overall prevalence of musculoskeletal pain in this sample was 62.4% and the two main significantly associated factors were walking time to school and relative weight of the school bag. A clear difference in mode of transport was observed between private (school bus) and governmental schools (walking), but the same factors remained significantly associated. These findings can aid formulating organisational recommendations for elementary schools in Ethiopia.

The prevalence of musculoskeletal pain found in the current study is similar to the results of studies done in Greece (64.2%) and India (63.2%) [6, 21]. Other studies, however, have found higher or lower prevalence rates. For example, a cross-sectional study done in Uganda [9] reported a prevalence of musculoskeletal pain of 88.8% in 532 students of 10–21 years old. A Brazilian cross-sectional study with a convenience sample of 262 children aged 6–12 years reported musculoskeletal pain to be present in 51.1% [22]. Possible reasons for different prevalence rates in these studies might be the sample size, age variation, environmental factors and facilities.

Walking to school with supplies for a longer period of time (heavy school bags and walking for 20 min) was found to be significantly associated with musculoskeletal pain. These findings are consistent with other studies which reported that long time walking with a school bag is significantly associated with back pain [6, 9, 12, 22, 23]. For example, a survey conducted in Australia reported that adolescents with long time walking with school supplies reported more musculoskeletal complaints than those who utilized transport facilities [24]. A study conducted in Uganda found that methods of carrying school supplies and long duration of walking were significantly associated with low back pain [9]. They found an Adjusted OR = 0.073 (95%CI 0.007 to 0.731) for those who carried school supplies in the hand compared to those carrying a hand bag with supplies, which can be explained (when converted as 1/0.073) as those who carried school supplies in the hand being 13.7 times more likely to experience musculoskeletal pain. Possible reason could be the position of upper extremities while holding the materials in the hand. As found in this study, heavy bags of more than 20% of body weight can also influence musculoskeletal pain. The interaction between heavier school bag weight in % of body weight and longer walking duration suggest that the children carrying heavier school supplies and at the same time walking longer have 3.5 times higher chances of developing musculoskeletal pain than those carrying lighter school supplies and/or walking lesser duration to or from school.

Other clear findings from this study with potential for organisational adjustments, is the difference between private and governmental schools with regards to the prevalence of musculoskeletal pain and mode of transport, and the facilities and physical (in)activity at schools. Walking to school appeared a significant associated factor for students of both governmental and private schools. The majority of private school children (77%) had motorised transport to school which could be a reason for the significantly lower prevalence of musculoskeletal pain in this group (50% versus 67%). A likely reason is the socio-economic status of families of school children attending governmental and private schools. Schools are recommended to provide transport services for school children living farther away from school.

Similarly, schools are recommended to provide locker facilities to store school supplies, such as books, to facilitate reducing school bag weight. Other factors such as poor ergonomic furniture, long sitting times and lack of varied physical activity through physical education should be taken into consideration by school authorities aiming to decrease musculoskeletal pain in their students.

### Study limitations

This study has provided well-powered insight into the prevalence, type of musculoskeletal pain and associated factors in school children in North-West Ethiopia. Although 94% of the calculated sample size was reached, post-hoc power analysis show 100% power was reached with the higher than anticipated prevalence. A few limitations can be mentioned to benefit future research. Schoolbag weight was recorded only once. Consequently, the recorded data did not account for the variance of school bag weight during a typical week. In addition, this study reported current pain and pain history rather than pain while carrying their school bag, which could lead to possible differences in estimation of the association between school bag weight and pain. Also, psychological factors, postural assessment and personal (home) factors were not considered. Personal factors could include help with household duties or farming. However, the results should be interpreted with caution because the findings are based on self-reported pain among children. The potential for social desirability bias due social attention seeking by children during pain reporting may occur. At same time it is difficult to attempt objective verification of pain among children. Nevertheless, the present findings indicate that transport specific interventions and school bag weight monitoring programs by schools is needed to reduce musculoskeletal pain.

## Conclusion

In conclusion, more than 60% of school children experienced musculoskeletal pain partly explained by the associated factors walking long distance to school and carrying heavy school bags. Other factors that may help explain musculoskeletal pain in children and should be explored in future studies. In the meantime, school authorities are recommended to provide transportation services to reduce the impact of long walking duration and provide locker facilities in schools for students to keep their school supplies in order to decrease weight, frequency and duration of carriage.

## Abbreviations

BMI: Body Mass Index
WHO: World Health Organization
